# Supporting the production of pharmaceuticals in Africa

**DOI:** 10.2471/BLT.15.163782

**Published:** 2015-11-26

**Authors:** Jicui Dong, Zafar Mirza

**Affiliations:** a Essential Medicines and Health Products Department, World Health Organization, avenue Appia, 1211 Geneva, Switzerland.

Ethiopia has become a model country for local investment in pharmaceuticals, as advocated by three United Nations agencies^1^ and the African Union Pharmaceutical Manufacturing Plan for Africa.^2^ Recently, Ethiopia launched a national strategy and plan of action to develop local pharmaceutical manufacturing capacity and increase access to locally manufactured, quality assured medicines.^3^ The World Health Organization (WHO), with support from the European Commission and the Bill & Melinda Gates Foundation, worked closely with the Government of Ethiopia in the development of this strategy.

Africa has more than 70% of the world’s people living with human immunodeficiency virus (HIV)/acquired immunodeficiency syndrome (AIDS) and 90% of the world’s deaths due to malaria; noncommunicable diseases are also an increasing problem.^4^ The demand for safe, effective and affordable medicines is therefore great, but weak local pharmaceutical manufacturing capacity means that Africa largely depends on imported medicines. It is estimated that around 79% of all pharmaceuticals in Africa are imported. This significantly increases health expenditure and leaves people vulnerable to interruption of the supply of medicines. Access to life-saving medicines is a human right, but it remains far from being guaranteed for the majority of people living in Africa. Long lead times in international procurement, fragile logistics and storage capacity, and high transport and distribution costs hinder the broad access and affordability of essential medicines. Moreover, patented medicines for diseases like hepatitis, cancer and multidrug-resistant tuberculosis may be out of reach for many patients because of their high cost.

Local production of quality-assured medicines is a critical strategy in the long term, in view of growing demands, as a way to ensure reliable access to affordable medicines. Africa is now the world’s second fastest growing pharmaceutical market, projected to reach 30 billion United States dollars per year by 2016.^5^ It is hoped that in Africa, as in other emerging economies, projected industrial growth and modernization can contribute to social development and catalyse knowledge-based economic growth, research and development.

Strong political commitment and leadership are needed to achieve this vision. In the case of Ethiopia, the government understands its role very well and is ready to lead. The successful transformation of the national pharmaceutical sector and the strengthening of pharmaceutical manufacturing capacity require that the government improve policy coherence in the pharmaceutical sector. Further support is now needed for human capital development, innovation, foreign direct investment, quality assurance systems and strengthening national regulatory authority. A responsive, outcome-oriented, predictable, risk-proportionate, and independent medicine regulatory authority is a prerequisite for building a competitive pharmaceutical industry and ensuring safe, quality-assured and efficacious medicine on the market.^6^ WHO and other international organizations have developed technical standards^7^ and offer technical assistance to developing countries in this respect. African countries interested in strengthening their pharmaceutical sector should encourage their pharmaceutical manufacturers to apply for WHO prequalification. National regulatory authorities also benefit from such developments.

A tailored package of incentives will encourage local pharmaceutical companies to survive fierce competition from the globalized pharmaceutical sector. Examples of such incentives include preferred public procurement, soft loans and non-fiscal facilitations e.g. development of cohesive policy frameworks, strengthening national regulatory authorities, support for capacity building, creation of an investment-friendly environment and infrastructure development. The level and duration of incentives have to be decided according to the situation in different countries.

Access to medicines is an integral component of universal health coverage. However, enhanced local production may not automatically lead to improved access to qualified medical products. Industrial development should be combined with national policy for universal health coverage so that local pharmaceutical production can address local health needs. A combined policy framework to identify potential common interests has been advocated by WHO.^8^ African countries have an opportunity to learn from, and avoid, problems experienced by other countries. India’s pharmaceutical industry has been successful as an exporter, but access to medicines has not always been improved for local communities. For example, first-line antiretroviral medicines are still not reaching many HIV/AIDS patients in India.^9^

As a result of cultural- and trade-related factors, Africa includes many different markets.^10^ The relatively small economic size of many African countries poses a challenge for pharmaceutical manufacturing. This could be addressed by regional market integration. Harmonization of the registration of medicines in Africa, supported by WHO, can play an important role in the long term development and sustainability of local production. Harmonization also makes good sense in view of the limited availability of qualified regulatory staff in the region.^11^

The Federation of African Pharmaceutical Manufacturers Associations (FAPMA) was inaugurated in 2013 with the mission:

“To facilitate collaboration between regional pharmaceutical manufacturing associations, to address the common challenges faced by the industry and to enhance opportunities towards self-sufficiency through advocacy and partnership with other stakeholders in promoting the production of quality, affordable medicines.”^12^

Progress has also been made in regional organizations. Effective regional partnership strategies suggest that borderless regional pharmaceutical industries might emerge and become new competitive operational entities in the African continent.^13^

Local pharmaceutical production in Africa has not yet received much support from the international community. There is a perception that Africa is not suitable for cost-effective production of quality-assured, safe medicines. Since locally produced medicines have to compete with low-priced Chinese and Indian generic products, some suggest that international procurement of inexpensive drugs is a more efficient use of funds, rather than investing in local industry.^14^ Mozambique and Zimbabwe were not able to produce medicines at competitive prices in the short-term; however, there may be long-term benefits from stimulating the development of local industrial complexes and secure national drug supply.^15^ Setting up local pharmaceutical production can also benefit associated local industries and businesses.

The determination demonstrated by Ethiopia in transforming its pharmaceutical industries and fostering local pharmaceutical production, along with the recent proliferation of similar initiatives across the continent, sends a clear message that African countries hold long-term visions, aim at integrated and sustainable development and expect national and continental cross-cutting modernization. In this context, international development partners should also find new ways of thinking in supporting the efforts of the African continent to eliminate poverty, disease and dependence.
